# Tetanus Following Menorrhagia with Purpura Hæmorrhagica

**Published:** 1875-10

**Authors:** 


Tetanus Following Menorrhagia with Purpura Hæmorrhagica—Hypodermic Injection of Chloral—Cure.—(*The Obstetrical Journal, August,* 1875.—Dr. Ribell relates the case of a patient, æt. 36, who suffered from purpura hæmorrhagica after each of her four confinements. Nine weeks after her last one, severe rigors set in, followed by contraction of the muscles of the neck, stiffness and difficulty of deglutition, with slight trismus. The symptoms increased rapidly, and left no doubt as to their nature. Fifteen-grain doses of chloral every half hour were given for three hours, when sleep supervened, and lasted five hours. The symptoms returned when the patient awoke, and gradually increased in severity, pains in the back and suffocation being complained of. Thirty grains of chloral in solution were injected into the side of the neck, and repeated every hour for six hours. Sleep then occurred, and lasted nine hours, the patient awakening free from all symptoms. Two hundred and ten grains in all were injected. Convalescence was slow.—*Medical Times.*

Inhalations of Wine of Ipecac Spray in Catarrh and Asthma. Drs. Binger and Murrel (*Gaz. Med. de Paris*, 1875, p. 374, from *Siglo Medico*) recommend this preparation, pure or diluted, according to the susceptibility of the subjects. The first liquid inhalation should be drawn deeply in by the mouth, the tongue being kept down and the nostrils closed. The earlier sittings should be short, with a period of rest between each three or four inspirations. Twenty-five patients have undergone this treatment at the hands of Drs. B. and M., of whom the majority were cured in twelve days, but a few before that time.

The first inhalations are sometimes followed by augmentation of the cough, dryness of the throat and hoarseness; these symptoms are only temporary. Between the respirations of the spray, gargles should be used to remove particles of ipecac from the pharynx, etc., so that nausea may be avoided. The number of sittings may vary from one to three a day, and the temperature, dilution, etc., may be changed to suit the individual case.—*Medical Times.*

